# Effect of nutritional and physical exercise intervention on hospital readmission for patients aged 65 or older: a systematic review and meta-analysis of randomized controlled trials

**DOI:** 10.1186/s12966-021-01123-w

**Published:** 2021-05-10

**Authors:** Ellisiv Lærum-Onsager, Marianne Molin, Cecilie Fromholt Olsen, Asta Bye, Jonas Debesay, Christine Hillestad Hestevik, Maria Bjerk, Are Hugo Pripp

**Affiliations:** 1 Lovisenberg Diaconal University College, Oslo, Norway; 2 Department of Nursing and Health Promotion, Faculty of Health Sciences, Oslo Metropolitan University, Oslo, Norway; 3 Department of Health, Bjorknes University College, Oslo, Norway; 4 Department of Physiotherapy, Faculty of Health Sciences, Oslo Metropolitan University, Oslo, Norway; 5 Regional Advisory Unit for Palliative Care, Department of Oncology, Oslo University Hospital, Oslo, Norway; 6 Norwegian Institute of Public Health, Oslo, Norway; 7 Faculty of Health Sciences, Oslo Metropolitan University, Oslo, Norway; 8 Oslo Centre of Biostatistics and Epidemiology, Research Support Services, Oslo University Hospital, Oslo, Norway

**Keywords:** Nutrition, Physical exercise, Older people, Readmission, Meta-analysis, Transitional care

## Abstract

**Background:**

Unplanned readmission may result in consequences for both the individual and society. The transition of patients from hospital to postdischarge settings often represents a discontinuity of care and is considered crucial in the prevention of avoidable readmissions. In older patients, physical decline and malnutrition are considered risk factors for readmission. The purpose of the study was to determine the effects of nutritional and physical exercise interventions alone or in combination after hospital admission on the risk of hospital readmission among older people.

**Methods:**

A systematic review and meta-analysis of randomized controlled studies was conducted. The search involved seven databases (Medline, AMED, the Cochrane Library, CINAHL, Embase (Ovid), Food Science Source and Web of Science) and was conducted in November 2018. An update of this search was performed in March 2020. Studies involving older adults (65 years and above) investigating the effect of nutritional and/or physical exercise interventions on hospital readmission were included.

**Results:**

A total of 11 randomized controlled studies (five nutritional, five physical exercise and one combined intervention) were included and assessed for quality using the updated Cochrane Risk of Bias Tool. Nutritional interventions resulted in a significant reduction in readmissions (RR 0.84; 95% CI 0.70–1.00, *p* = 0.049), while physical exercise interventions did not reduce readmissions (RR 1.05; 95% CI 0.84–1.31, *p*-value = 0.662).

**Conclusions:**

This meta-analysis suggests that nutrition support aiming to optimize energy intake according to patients’ needs may reduce the risk of being readmitted to the hospital for people aged 65 years or older.

**Supplementary Information:**

The online version contains supplementary material available at 10.1186/s12966-021-01123-w.

## Background

Unplanned readmission implies consequences both for the individual and society [1]. On the individual level, readmission and hospitalization is associated with poorer health, reduced physical function, malnutrition and reduced quality of life [1–3], while the societal consequences include pressure on the health-care system and increased hospital costs [4]. The 30-day readmission rate is used as a quality measure and it has been reported that up to one quarter of all hospital admissions are readmissions in the US [1]. In Western Europe, such as Denmark and Norway, readmission rates have been reported to be 18 and 16% respectively [5, 6]. To prevent or delay these consequences, it is important to identify interventions that may reduce unplanned hospital readmissions in populations transitioning from one care setting to another [7].

Several studies have suggested that interventions, including physical exercise and nutrition, may improve general health in older adults [8–11]. A meta-analysis investigating physical exercise-based interventions in older patients concluded that such interventions may improve functional ability and, therefore, prevent readmission to the hospital [12]. This study showed the most positive results in interventions that included in-hospital and postdischarge components. However, another meta-analysis of 12 randomized controlled trials (RCTs) that included older patients ≥65 years found no effect on readmission rates of interventions that combined physical exercise and various educational components, such as coping strategies for disease-related symptoms [13]. Regarding nutrition, a previous study found that almost 40% of hospitalized elderly patients were malnourished [14] . A meta-analysis aiming to investigate the effects of interventions to improve the nutritional status of patients with a mean age ≥ 65 years found that supplementation with oral nutritional supplements (ONS) during and following hospital stay resulted in a significant reduction in hospital readmissions [15]. Another meta-analysis investigating the effect of a multidisciplinary nutritional approach on hospital readmissions in the same age group found no significant effects of the intervention [16].

Aiming to improve physical function or nutritional status to reduce readmission rates seems reasonable due to physical decline during and after hospital admission and a high prevalence of malnutrition during hospitalization among older patients. However, there is currently limited knowledge on whether physical exercise and/or nutritional interventions following or during a hospital stay will have any effect on hospital readmission. Thus, this systematic review aimed to review and synthesize the literature to make a comprehensive up-to-date meta-analysis of randomized controlled trials on the effect of physical exercise and nutritional interventions on hospital readmissions for older patients.

## Methods

The Preferred Reporting Items for Systematic Reviews and Meta-Analyses statement was used to structure this systematic review and meta-analysis [17]. The protocol for this systematic review and meta-analysis was registered in PROSPERO (registration number: CRD42020154724).

### Information sources and search strategy

A systematic literature search for articles published up to November 2018 was conducted by four research librarians using seven databases: Medline, Embase (Ovid), the Allied and Complementary Medicine Database, the Cochrane Library, the Cumulative Index to Nursing and Allied Health Literature via EBSCO, Food Science Source and Web of Science. In addition, an updated literature search was performed in March 2020. The literature searches included the following elements: older patients, readmission, nutrition and physical activity (see Additional file 1 for the full electronic search strategy for all the databases). There were no limits for date of publication, language and setting for the intervention—that is, interventions could be carried out post discharge and in outpatient clinics, nursing homes and hospitals.

### Study selection

The titles and abstracts of all articles retrieved in the initial search were evaluated independently by pairs of two or three authors (AB, CFO and JD; CH and MB; and ELO and MM). All the retrieved articles were reviewed in accordance with the inclusion and exclusion criteria. The updated search was reviewed by two of the authors (ELO and MM). The reference lists of the retrieved studies were examined for relevant studies. Any disagreements regarding the inclusion or exclusion of articles were discussed and resolved by consensus between the authors.

### Eligibility criteria

The following inclusion criteria were employed: a) RCTs; b) enrolled patients ≥65 years; c) interventions consisting of physical exercises and/or nutrition aimed at optimizing the energy intake according to the patients’ needs, such as ONS, improved nutritional care or dietary counseling; and d) the report of hospital readmission at ≤90 days as an outcome. The exclusion criteria included a) all other study designs (excluding RCT) and b) multimodal or multicomponent interventions, defined as studies including more than two interventions, and nutritional interventions consisting of only dietary supplements, parenteral and/or enteral nutrition or fluid restriction.

### Data collection and extraction

Data extractions were performed by pairs of two or three authors (AB, CFO and JD; CH and MB; and ELO and MM) and entered into a predefined data extraction table. The following information was extracted: first author, publication year, country, study setting, sample size, sex, age, type of intervention (i.e., nutrition, physical exercise activity or both), duration of intervention, number of events of readmission and readmission duration. If needed, we contacted the authors to receive additional data not reported in the original articles, e.g. the age range for the total sample.

### Risk of bias

Articles considered for inclusion after the second evaluation were independently assessed for methodological quality by two authors (ELO and MM). The Cochrane Risk of Bias Tool version 2 (RoB 2) was used to assess the quality of the RCT studies [18]. RoB 2 includes five domains of bias: bias arising from the randomization process, bias due to deviations from the intended interventions, bias due to missing outcome data, bias in the measurement of the outcome and bias in the selection of reported results. In addition, RoB 2 includes a domain regarding the “overall risk of bias.” Each domain consists of different signaling questions, and the risk-of-bias judgments for each domain are based on the answer to the signaling questions and can be classified as “low risk of bias,” “some concerns” or “high risk of bias” [18]. Disagreement regarding the individual judgment of each of the domains was resolved by consensus between the two reviewers.

### Statistical analysis

Study heterogeneity was assessed by examining the data extraction tables. The authors considered study designs, population characteristics and interventions and determined if they were satisfactorily homogeneous to permit a meta-analysis. Based on this, a meta-analysis with risk ratio (RR) as effect size and the Mantel-Haenzel method to estimate the pooled effect size across studies was considered appropriate. In addition, we presented the pooled effect size from the fixed-effect inverse-variance method. We calculated RR, with 95% CIs and *p*-values, to assess pooled readmission risk and I^2^ statistics and Q-statistics with p-value and degree of freedom to assess statistical heterogeneity and consistency [5, 19]. Publication bias was assessed using funnel plots and Egger’s test for small-study effects. Stata version 16 (Stata Corp, Texas, USA) was used with the meta-analysis packages metan [20], metafunnel [21] and metabias [22] packages for all the estimations.

## Results

### Literature search and study characteristics

**Fig. 1:**
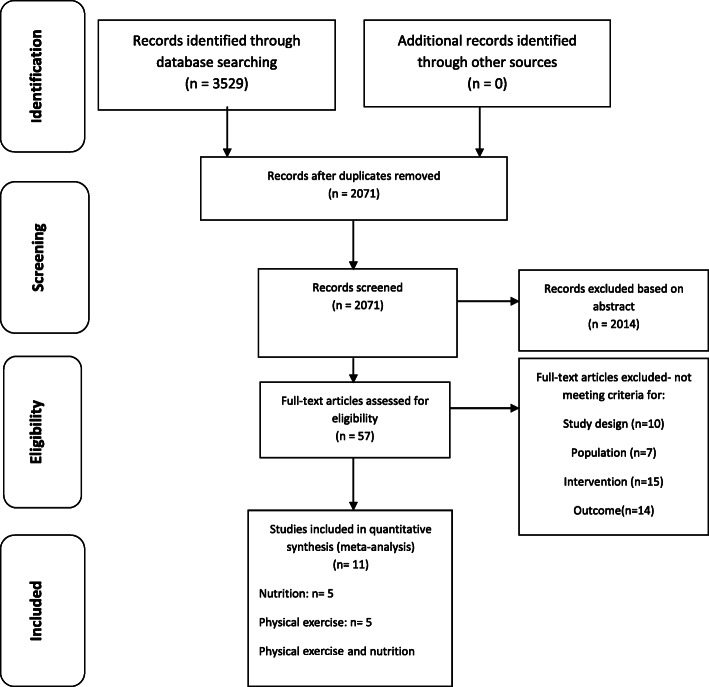
Summary of literature search and selection of articles

The search produced 2071 studies, which resulted in 1458 studies after the removal of duplicates. Of these, we identified 57 full-text studies that were assessed for eligibility, of which 11 studies were assessed for quality and included in this systematic review and meta-analysis. A flowchart describing the process of study selection is presented in Fig. 1 [23].

The included studies were conducted in Australia (*n* = 4) [24–27], Denmark (*n* = 2) [28, 29], Ireland (*n* = 1) [30], Spain (*n* = 2) [31, 32] and the US (*n* = 2) [33, 34] (see Table 1). The sample size ranged from 100 to 652 participants, with a total of 2681 participants. Most of the participants were female (53–83%), and the mean age ranged from 71.1 (SD: 7.95) [34] to 88 (SD: 5) [31]. In all the included studies, the participants were recruited on hospital admission [24–34], and the interventions were carried out in the patient’s home after discharge (*n* = 1) [34], at the hospital and at home (*n* = 5) [25, 27–29, 33] or only at the hospital (*n* = 5) [24, 26, 30–32].

**Table 1:** Characteristics of the reviewed studies

Study	Country	Study setting	Sample size	Sex (N, %)	Mean age (SD, range)	Type of intervention	Readmission duration
Deer et al. 2019 [34]	United States of America	Recruitment: Patients admitted to hospital for acute illness. Intervention: at home	100	M: 30 (30) W: 70 (70)	71.1 (SD =7.95)	C	30 days
De Morton et al. 2007 [24]	Australia	Recruitment and intervention: general medical wards at one public acute, secondary and tertiary hospital	236	M: 107 W: 129	78 (7): control group 80 (8): intervention group	P	28 days
Finlayson et al. 2018 [25]	Australia	Recruitment: Medical wards at two tertiary metropolitan hospitals Intervention: home	222	M:60 (27) W: 162 (73)	77.6 (SD = 6.64, range: 65–93 years)	P	28 days** 84 days
Martínez-Velilla et al. 2019 [32]	Spain	Recruitment and intervention: acute care unit at one tertiary public hospital	370	M: 161 (43) W:209 (57)	87.3 (SD = 4.9, range: 75–101)	P	84 days
McCullagh et al. 2020 [30]	Ireland	Recruitment and intervention: all wards at a general teaching hospital	190	M:90 (47) W:100 (53)	80.0 (SD = 7.5, range: 65–97)	P	84 days
Ortiz-Alonso et al. 2020 [31]	Spain	Recruitment and intervention: acute care for elders’ unit in a Public Hospital	268	M: 115 (43) W:153 (57)	88 (SD = 5, range: 75–102)	P	84 days
Holyday et al. 2012 [26]	Australia	Recruitment and intervention: two acute geriatric medicine wards at one hospital	143	M:61 (43) W:82 (57)	^a^	N	30 days
Deutz et el. 2016 [33]	United States of America	Recruitment: 78 hospitals Intervention: hospital and home	652^b^	M:298 (46) W: 354 (54)	78.1 (8.6): placebo group 77.7 (8.2): intervention group	N	30 days 60 days 90 days**
Lindegaard Pedersen et al. 2017 [28]	Denmark	Recruitment: Department of Geriatrics at one university hospital Intervention: hospital and home	208	M: 35 (17) W:173 (83)	86.1 (SD = 5.64), range 75–103)	N	30 days** 90 days
Sharma et al. 2017 [27]	Australia	Recruitment: General medicine department in a public hospital Intervention: hospital and primary healthcare sector after discharge	148	M:54 (36) W:94 (64)	81.8 (SD = 8.7, range: 60–97)	N	30 days 84 days
Terp et al. 2018 [29]	Denmark	Recruitment: Department of geriatric medicine in a university hospital Intervention: hospital and primary health sector after discharge	144^c^	M: 31 (23) W:113 (77)	87 (SD = 6)	N	90 days

*C* combination of nutrition and physical exercise intervention

*P* physical exercise intervention

*N* nutrition intervention

** readmission is primary outcome

^a^ = author has not responded to data request

^b^ = all patients were malnourished at inclusion according to SGA

^c^ = all patients were malnourished at inclusion according to NRS-2002

Readmission was the primary outcome in three studies [25, 28, 33], and the registration period for readmission varied between 28 days [24, 25] and 90 days [28, 29, 33]. The interventions included physical exercise (*n* = 5) [24, 25, 30–32], nutrition (*n* = 5) [26–29, 33] and a combination of nutrition and physical exercise (*n* = 1) [34].

### Physical exercise interventions and readmission

Five of the included RCTs investigated the effect of exercise intervention on readmission. All the exercise programs consisted of strength exercises. One study combined strength with balance exercises [34], and four studies employed programs with a combination of strength, balance and walking [24, 25, 30, 32] (see Table 2). The dose of the exercise interventions varied. The duration of each exercise session was reported to be 20–30 min in four studies [24, 30, 32], up to 2 h in one study [25] and in the study combining physical activity and nutrition duration was not reported [34]. Regarding frequency, one study reported that participants exercised 3 days a week for 4 weeks [34], (12 sessions), while three studies [24, 30, 32] reported that participants exercised twice daily during hospital admission. The exercise programs were individually tailored, in terms of exercise intensity, to each participant in all the studies. Only one study refers to a specific intensity measurement; namely 30% of one repetition maximum [32]. In three of the studies, physiotherapists prescribed and supervised the exercises [24, 30, 34], while two studies used fitness specialists [31, 32] and one exercise physiologist [25]. Regarding setting, three studies applied the exercise intervention during hospital admission only [24, 30, 32] and one in-home only [34]. One study started the exercise program in the hospital and continued at home for 24 weeks post discharge [25]. The total duration of the interventions varied. The shortest duration reported was by Ortiz-Alonso et al. [31], with a median of 3 days during hospital admission, and the intervention with the longest duration was the intervention by Finlayson et al. [25]. Two studies did not report the number of intervention days [24, 30]. Finally, adherence to the exercise intervention varied between 95.6% [32] and 42% [25].

**Table 2:** Description of intervention, length of follow-up and adherence to the intervention organized according to intervention and year of publication

Study	Intervention type	Description of intervention	Length of intervention	Intervention adherence	Completion rate
**Deer et al, 2019** [34]	Combination of physical exercise and nutrition	**Physical exercise intervention**: Progressive in-home rehabilitation training program 3 days a week. The program was prescribed and overseen by a physical therapist and supervised one to two times a week by research staff with the remaining exercise session(s) performed without supervision. Exercises included chair rises, toe stands and three seated exercises using TheraBands; knee extension, rows and arm extensions. The exercise was designed to begin at low to moderate intensity and to be progressed during the 4 weeks by changing the resistance of the Theraband. The exercise was given either alone or in combination with 20 g whey protein twice daily. **Nutrition intervention:** participants were instructed to take 20 g whey protein (22 g BiPro; Eden Prairie, MN) mixed with 8 oz. of water twice a day (morning and evening). The protein supplement was given alone or in combination with the physical exercise intervention. **Arms:** Two arms; 1.placebo (20 g maltodextrin twice daily, isocaloric to protein), 2. testosterone given as a single injection	Four weeks	Physical exercise adherence 77%Supplement adherence 75%	79/100
**Finlayson et al., 2018** [25]	Physical exercise	**Intervention**: Hospital physiotherapist assessed the patient and designed tailored exercise programs (taking approximately 2 h) designed to improve strength, stability, coordination, endurance, mobility, and improve self-confidence with respect to ADL. The exercise prescription was developed using a team approach involving the patient, caregivers, doctors, and ward nurses. Goals were defined for each patient and used as a motivational strategy to improve compliance with the program. After discharge: participants exercise on their own and received six-weekly in-home follow-up visits by an exercise physiologist requiring about 2 h pr visit. Here, support was offered together with reinforcement and further explanation of the exercise program. **Arms:** Three arms: 1. Exercise only 2.Nurse Home visits and Telephone follow-up (N-HaT) 3. Exercise program and Nurse Home Visit Telephone follow-up and (EXN-HaT) **Controls**: Usual care	In hospital and 24 weeks after discharge	Adherence to the home-based exercise over the 24 weeks varied between 42 and 68%.	183/222
**Martinez-Velilla et al., 2018** [32]	Physical exercise	**Intervention**: two daily sessions (morning and evening) 20 min during 5 to 7 consecutive days. An experienced fitness specialist supervised each session. Exercises were adapted from the multicomponent exercise program called *Vivifrail* [35] to prevent weakness and falls. Morning sessions included individualized supervised progressive resistance, balance and walking training exercises. The resistance exercises were tailored to the individual’s functional capacity using variable resistance training machines aiming at 2–3 sets of 8 to 10 repetitions with a load of 30–60% of 1 repetition maximum. 3 exercises mainly involved lower limbs and 1 involved upper body muscles. Balance exercises and walking exercises were gradually progressed in difficulty. Evening sessions consisted of functional unsupervised exercises using light loads (anklets and handgrip balls) such as knee extension and flexion as well as daily walks in corridor. **Control:** usual care	During hospital stay/admission(mean no of intervention days: 5)	Adherence varied between 95.8% for the morning sessions and 83.4% for the evening sessions	310/370
**McCullagh et al., 2020** [30]	Physical exercise	**Intervention**: augmented prescribed exercise program (APEP). Up to 30 min exercises twice daily. Tailored exercises to improve strength, balance and walking supervised by a physiotherapist Monday-Friday. **Control:** Sham exercise program up to 30 min twice daily assisted by a physiotherapist; breathing and stretching exercises	During hospital stay/admission (median length of stay: 8 nights)	Adherence, defined as completed ≥75% of possible exercise sessions, was 66% of participants in the intervention group, and 60% in the control group	145/199
**De Morton et al., 2007** [24]	Physical exercise	**Intervention**: Usual care and individually tailored exercise program designed by a physiotherapist consisted of upper limbs and lower limbs, and trunk exercises. It included four exercise levels; 1: bed exercises 2 sitting exercises 3 standing exercises 4 stair exercises. Gravity, body weight and light weights were used for resistance when possible. Resistance increased when participants could perform 10 repetitions. Participants exercised for 20–30 min sessions, twice daily, 5 days a week supervised by a certified allied health assistant. **Control:** usual care only	During hospital stay/admission (median length of stay: 5–6 days)	A	167/236
**Ortiz-Alonso et al. 2020** [31]	Physical exercise	**Intervention**: In addition to usual care, the exercise started the day after admission, was performed on weekdays and included 1 to 3 sessions per day (total duration, ca 20 min/day). It consisted of 1) rising from a seated to an upright position (using armrests/assistance if necessary) in the patient’s room (from 1 to a maximum of 3 sets of up to a maximum of 10 repetitions for each session; 2-min rest between sets and 2) 3–10 min of supervised walking on the corridor, using assistance (mobility aids such as walkers, or an external person) if needed. Standing and walking exercises were separated by a rest period of up to 5 min. The exercises were individually adjusted and supervised by a fitness specialist. **Control:** usual care only	During hospitalization (median length of stay:7 days, median training days: 3)	Median 3 training days and 2 training sessions per day.	268/281
**Holyday et al 2012** [26]	Nutrition	**Intervention:** Nutritional screening when hospitalized. Patients confirmed at nutritional risk referred to a dietician. Individualized nutrition intervention aiming to meet energy and protein requirements (ONS, snacks, texture modification and fortification, assistance with meals by ward staff, education of patients and carers, referral for discharge planning). **Control:** Nutritional screening when hospitalized. Ward not informed about screening result and occasional referral to a dietician.	During hospitalization	A	A
**Deutz et al 2016** [33]	Nutrition	**Intervention:** Standard nutritional care (usual care) and daily two servings of high energy and protein ONS containing beta-hydroxy-beta metylbutyrat (HMB), during hospitalization and 90 days after discharge. **Control:** Usual care and a placebo ONS twice daily.	During hospitalization/30, 60 and 90 days after discharge	Mean ONS per day: 30 days, intervention 1.65 (*n* = 242), control 1.69 (*n* = 227) 90 days, intervention:1.54 (*n* = 243), Control: 1.57 (*n* = 231)	622/652
**Lindegaard Pedersen et al 2017** [28]	Nutrition	**Intervention:** Standard nutritional care during hospitalization including estimation of energy and protein needs, nutritional therapy and recording of food and fluid intake and discharge arrangements (meal service, food delivery, home care). After discharge individualized counselling and follow-up (1, 2, and 4 weeks after discharge) by a dietician (home visit or by telephone) The patient’s home carer attended the home visits. Patients were encouraged to take active part in their own nutritional care. **Control:** Standard nutritional care during hospitalization, no follow-up.	During hospitalization/30 and 90 days after discharge	80% received three visits, 6% of the home carer attended three home visits.	75/117
**Sharma et al 2017** [27]	Nutrition	**Intervention:** Nutritional screening and referral to a ward dietician immediately after confirmed nutritional risk, when hospitalized. Individualized nutrition intervention initiated within 24 h upon referral aiming to meet energy and protein requirements (ONS (1–2.2 kcal/ml and 0.05–0.12 g of protein/ml), mid-meal snacks, food fortification, assistance with meals by ward based staff). Follow-up after discharge monthly by telephone. **Control:** Nutritional screening and referral to a dietitian by their treating clinicians (usual care). Patients at nutritional risk received the same intervention as the intervention group, but no follow-up after discharge.	During hospitalization/30 and 84 days after discharge	73% adherence at 1 month and 77% at 2 months. Forty-three (61.4%) control patients received dietitian input during hospital admission with no post-discharge outpatient dietetic follow-up.	103/148
**Terp et al 2018** [29]	Nutrition	**Intervention:** Nutritional screening and referral to a dietician when needed during hospitalization. Individualized counselling resulting in dietary plan for home, including pre-discharge advice on nutritional intake, combined with three follow-up visits by one trained person from the municipality after discharge (1, 4, and 8 weeks). Prescription of oral nutritional supplements (ONS). **Control:** Usual care during hospitalization (nutritional screening and referral to a dietician when needed).	During hospitalization/90 days after discharge	60% received three visits, 19% received no visits	103/150

*A* not reported

**Fig. 2:**
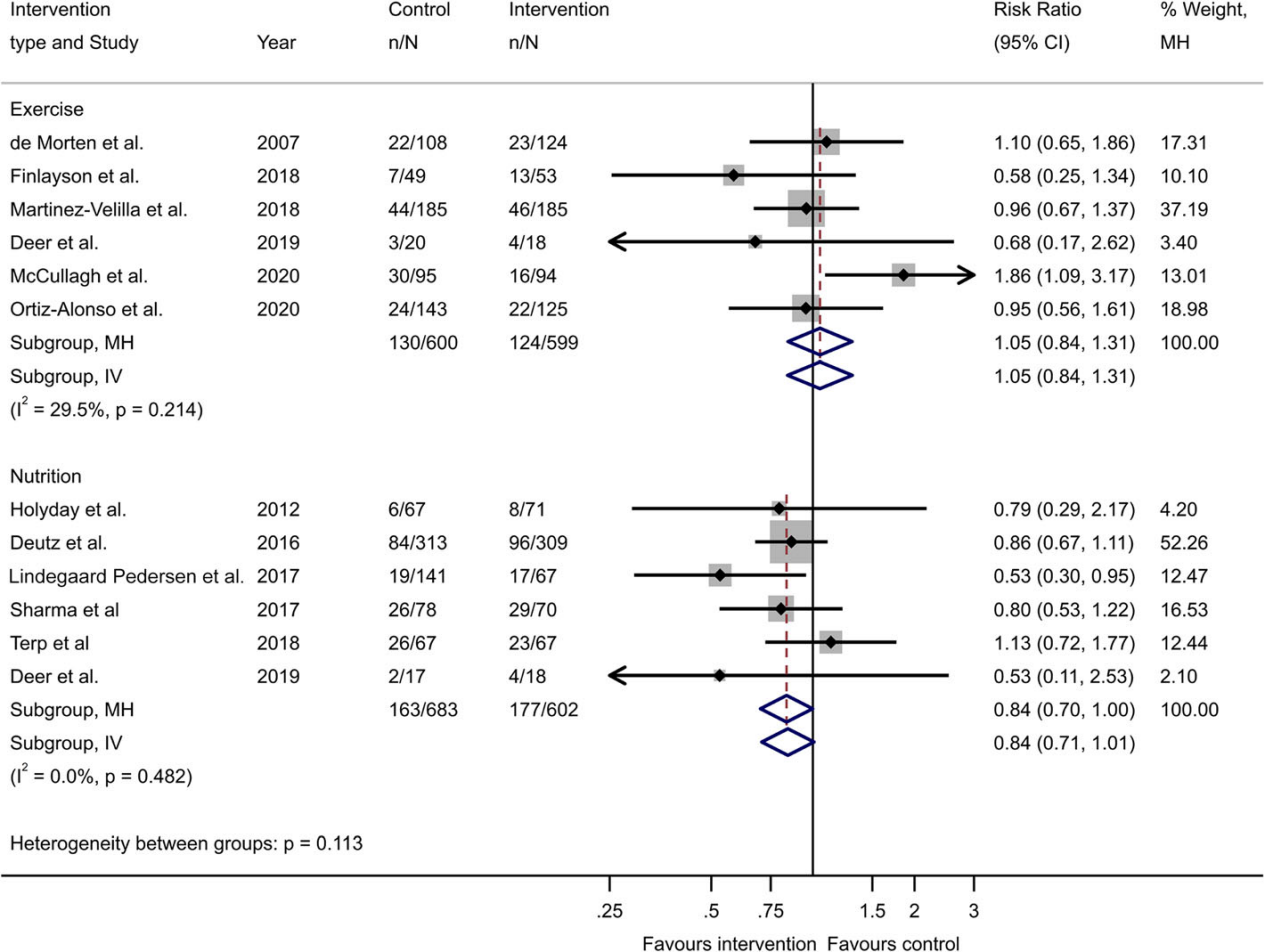
Pooled analysis presented as forest plots of the included studies on physical exercise and nutrition

**Fig. 3:**
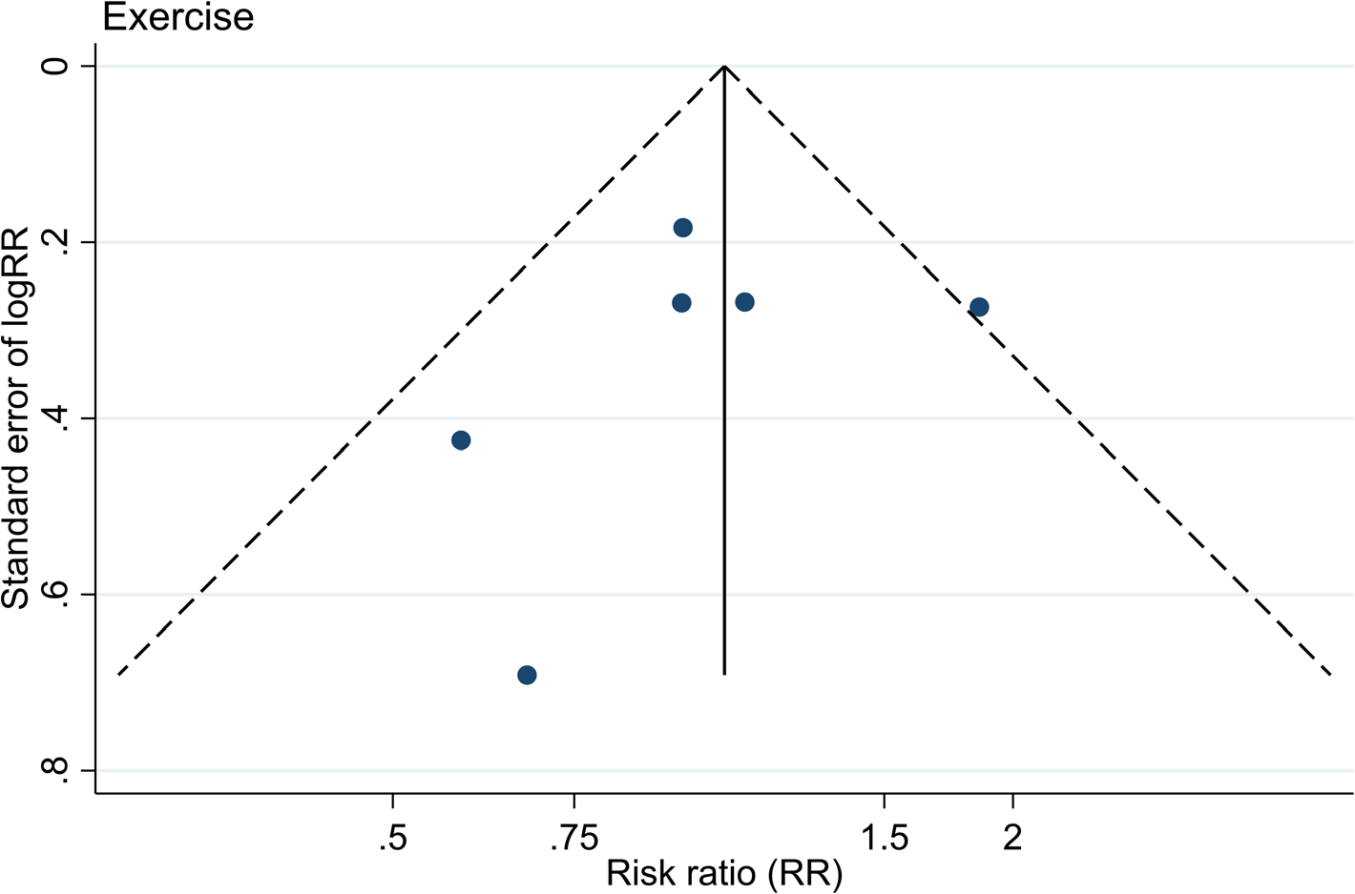
Funnel plot of the included studies on physical exercise

A pooled analysis from the six included studies on physical exercise intervention is presented in a forest plot with a pooled RR of 1.05 (95% CI 0.84 to1.31, *p*-value = 0.662; Fig. 2). The heterogeneity was low, with a corresponding I^2^ statistics value of 29.5% and the Mantel-Haenszel Q-statistics for heterogeneity of 7.09 (*p*-value = 0.214). Further, the funnel plot did not indicate any strong publication bias (Fig. 3), and Egger’s test for a small-study effect was insignificant (*p*-value = 0.592). The pooled RR from the fixed-effect inverse-variance was similar (RR 1.05, 95% CI 0.84 to 1.31, *p*-value = 0.669).

### Nutritional interventions and readmission

All nutrition interventions involved individualized dietary counseling based on regular food [28, 29], the use of ONS [33, 34] or a combination of these interventions aiming to meet estimated individual needs for energy and protein [26, 27] (see Table 2).

The interventions were need-based—that is, they were initiated after a screening for nutritional risk at the hospital. In most of the studies, registered dieticians performed the assessment and delivered the interventions [26–29], while in two studies, health-care personnel in the ward assessed and delivered the interventions [33, 34]. In five out of the six studies, patients after discharge received follow-up visits at home or were contacted by telephone [27–29, 33, 34]. The patient’s home carer was involved in both counseling and follow-up visits in two of the studies [26, 28]. In one study, a trained person from the municipality was responsible for the follow-up visits after discharge [29]. In Deutz et al.’s [33] and Deer et al.’s [34] studies, the nutritional intervention included ONS. Deutz et al. [33] used two servings of high energy and protein ONS containing beta-hydroxy-beta-methylbutyrate, a metabolite of leucine believed to induce anticatabolic, anabolic and lipolytic effects. Deer et al. [34] instructed the participants to take whey protein supplements consisting of 20 g whey protein mixed with 8 oz. water twice daily, morning and evening. In two of the studies, all participants were at nutritional risk, according to Subjective Global Assessment (SGA) [33] and Nutritional Risk Screening 2002 (NRS-2002) [29]. The interventions lasted up to 90 days. Adherence to the interventions was described in five of the six nutritional studies [27–29, 33, 34]. Four studies reported adherence as attendance to the home visits, which varied between 60 and 80% [27–29, 34]. One study reported adherence as consumption of the planned amount of ONS (median 95% at 10 days post discharge and 90% at 30 days) [33].

**Fig. 4:**
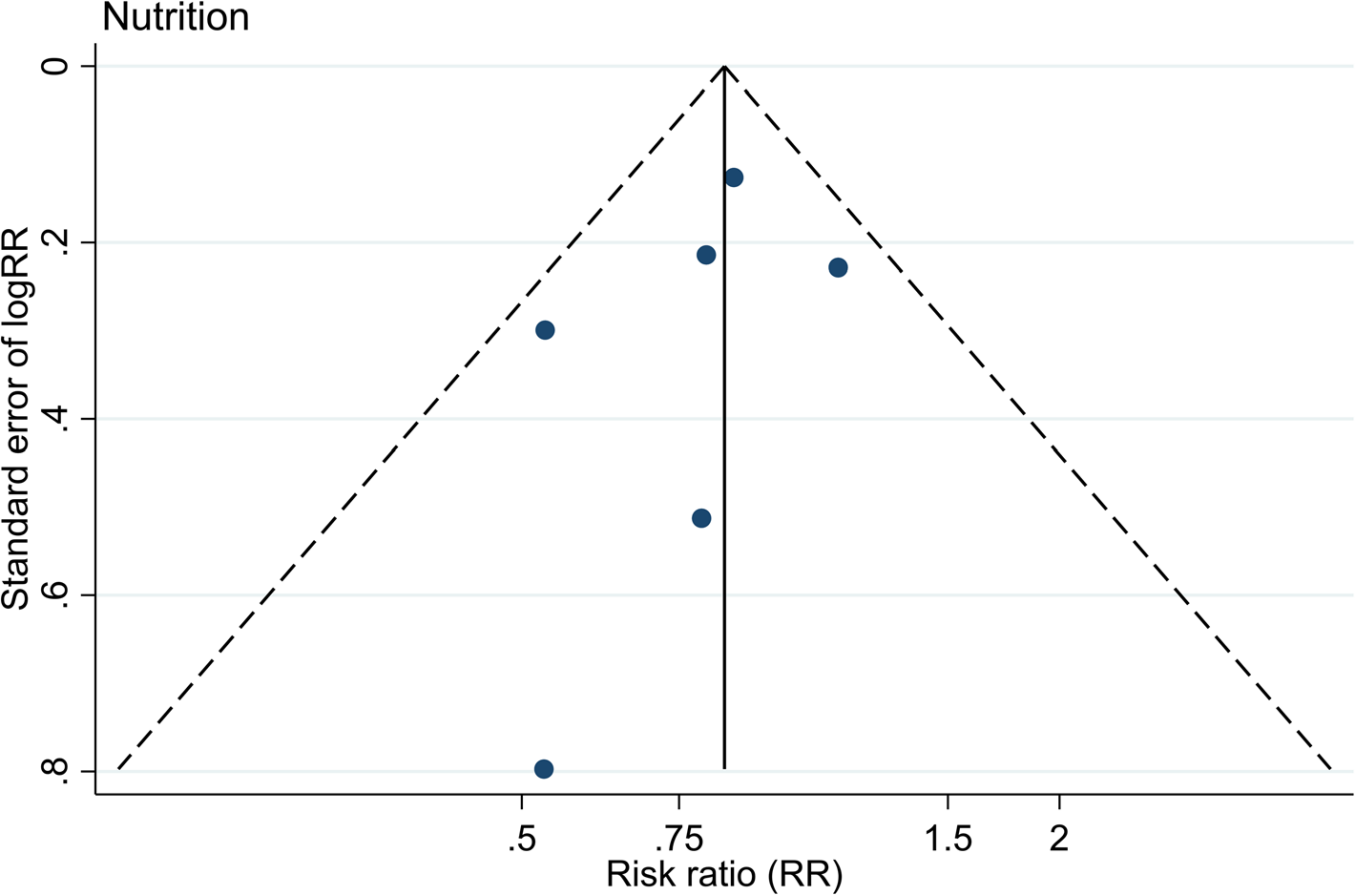
Funnel plot of the included studies on nutrition

A pooled analysis of the data from nutritional interventions is presented in a forest plot showing a pooled RR of 0.84 (95% CI 0.70– to 1.0, *p*-value = 0.049; Fig. 2). The heterogeneity was low, with an I^2^ statistics value of 0.0% and the Mantel-Haenzel Q-statistics for heterogeneity of 4.49 (degrees of freedom = 5, *p*-value = 0.482). Further, the funnel plot did not indicate any strong publication bias (Fig. 4), and Egger’s test for a small-study effect was insignificant (*p*-value = 0.440). However, the pooled RR using the fixed-effect inverse-variance method was not statistically significant (RR = 0.84, 95% CI 0.71 to 1.01, *p*-value = 0.061).

### Risk of bias

**Fig. 5:**
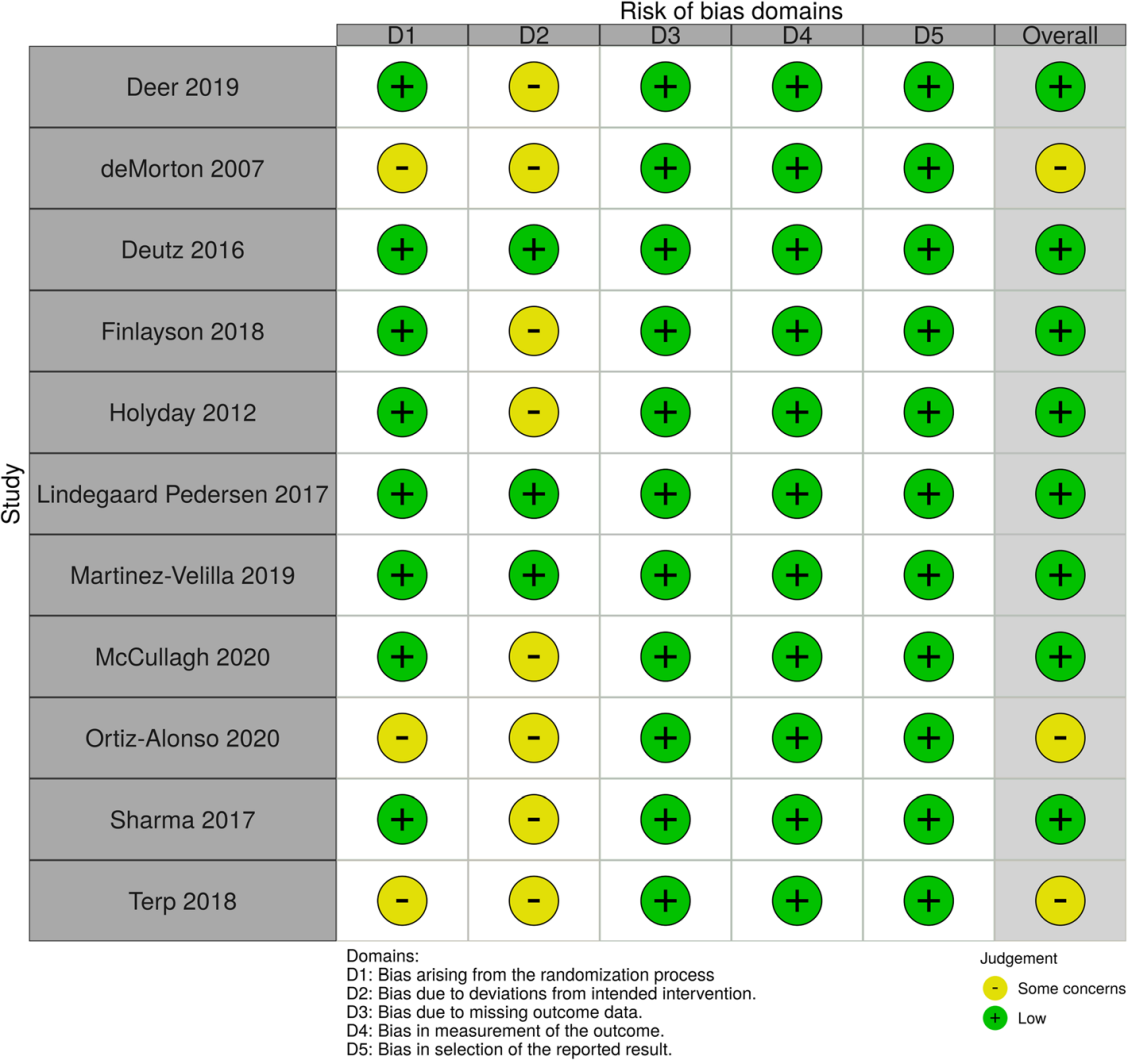
Methodological quality of the studies (Risk of bias)

Figure 5 shows the methodological quality of the studies [36]. In summary, three of the studies were rated as “some concerns” for bias arising from the randomization process, while eight studies were rated as “some concerns” for bias due to deviations from intended interventions. Bias due to missing outcome data, bias in measurement of the outcome and bias in selection of the reported results were rated as “low” for all studies. The overall risk of bias was rated as “some concerns” for three studies. In total, three studies were rated as “low risk of bias” for all five domains.

## Discussion

This is the first systematic review and meta-analysis that examines the effect of both physical exercise interventions and nutritional support strategies (nutritional counseling and/or supplementation with ONS) on unplanned readmission among older persons above 65 years. Interventions aiming to improve physical function with physical exercise during and/or after a hospital stay did not have any statistically significant impact on hospital readmissions, while the findings suggest that nutritional interventions during hospitalization and/or after discharge may reduce readmission.

### Effect of physical exercise

Overall, the physical exercise studies showed low statistical heterogeneity, which indicates a high consistency of effects across studies [19]. However, as remarked by Thompson [37], authors should also consider clinical heterogeneity (e.g., differences in intervention characteristics, such as dose and frequency of dose) among studies. Clinical heterogeneity in studies can cause inaccurate conclusions and further mislead decision-makers and health personnel [38]. Five of the studies included physical exercise interventions combining endurance, balance, flexibility and/or resistance training [24, 25, 30–32]. Previous studies have suggested that such multicomponent physical exercise training, preferably including all aforementioned components, has a positive effect on disability, functional ability and other health outcomes in frail older adults [39, 40]*.* Furthermore, the majority of the interventions involved exercise interventions of short duration and only during hospital stay [24, 30, 32]. According to a systematic review investigating the effectiveness of exercise interventions in frail older patients, interventions lasting longer than 5 months seem to have a more positive impact on health outcomes than shorter duration interventions [40]. Thus, overall, the duration of the interventions in the present meta-synthesis may be too brief to improve physical function in a manner that reduces hospital readmission.

Another factor that might have affected the benefits obtained in the different outcomes could be the intensity of the exercises, and a certain threshold of intensity might be necessary [41, 42]. Only one of the included studies reported intensity [32], and none of the studies discussed the ideal intensity of the physical exercise programs for older hospitalized patients. In general, a higher intensity is correlated with greater improvement in health outcomes compared to a lower intensity, but it is also suggested that a higher intensity is associated with potentially poorer adherence, especially in older patients [42]. Four of the six studies reported adherence to the exercise interventions, which varied considerably. In the physical exercise studies, Dishman [43] described adherence above 50% as acceptable. When this criterion is used for the included studies, three of the studies can be characterized as having an acceptable-to-high adherence [30, 32, 34], while one study have low adherence [25].

Our findings indicate that the physical exercise interventions did not have a statistically significant impact on hospital readmission in older patients. This finding is supported by another meta-analysis investigating multicomponent interventions, including early rehabilitation, in acute geriatric patients [44]. They also found no significant difference in hospital readmission within 1 or 3 months of hospital discharge between the groups receiving multicomponent intervention and the control group [44]. Nevertheless, the effect of physical exercise in older patients has been extensively and systematically investigated, and physical exercise has been shown to have a positive effect on cognitive and physical function [45, 46], quality of life [47] and sleep quality [48], as well as fall prevention [49]. Thus, although we did not detect any statistically significant effect in our meta-analysis, it is reasonable to suggest that the effect of physical exercise on the aforementioned factors may indirectly affect readmission in a positive manner.

### Effect of nutrition

The results from the meta-analysis show that patients receiving nutritional interventions had 16% less risk of being readmitted to hospital compared to the patients that received standard care. Due to the high prevalence and negative consequences of malnutrition in older hospitalized patients, it is important to investigate effective measures to prevent and treat this medical condition [14, 50]. Our results indicate a statistically significant reduction in risk for readmission, but the clinical relevance of the reduction is unclear. However, the statistical significance of nutritional interventions is uncertain and depends on the method to pool effects across studies. Nevertheless, due to older patients’ high readmission rates, even a small reduction is important for the patients, hospital workload and health economy [6].

Our results are in line with a previous meta-analysis, which concluded that supplementing with ONS in the hospital and/or in a community setting after discharge from the hospital reduced readmissions [15]. In our study, the most frequent nutritional intervention was individualized dietary counseling. According to the European Society for Clinical Nutrition and Metabolism guideline for clinical nutrition and hydration in geriatrics, individualized dietary counseling is considered the first line of nutrition therapy [51]. Individual counseling may include written advice, telephone contact, education sessions and all other forms of nutritional therapy [51]. One previous meta-analysis investigating the effect of individual dietary counseling provided by a dietitian in older patients at nutritional risk concluded that such counseling could improve nutritional status, but due to the lack of data, they could not investigate the effect of nutritional counseling on readmission [52].

One may consider whether nutritional counseling should be given to all older patients rather than be need-based. Need-based intervention is in accordance with existing guidelines and was applied in Munk et al.’s [52] study and this meta-analysis. However, different screening tools and assessment methods were used to identify patients’ nutritional status—that is, the Mini Nutritional Assessment [26, 28], NRS-2002 [29], SGA [27, 33] and BMI and dual-energy X-ray absorptiometry. Also, two studies exclusively included older patients at nutritional risk according to SGA [33] and NRS-2002 [29], while the respondents in the three other studies were screened for nutritional risk after inclusion. The use of different screening tools and assessment methods, as well as differences in inclusion criteria (only patients at nutritional risk vs. all patients), can affect the results. No international consensus exists regarding which nutrition screening tool has the best psychometric properties. According to a systematic review investigating nutritional screening tools used in hospital settings, none of the most commonly used tools, including the Mini Nutritional Assessment, NRS-2002 and SGA, performed consistently well for assessing patients’ nutritional status or predicting poor nutrition-related outcomes [53]. Furthermore, Munk et al. [52] underscored the importance of employing a nutritional screening tool that has predictive validity in order to identify patients who will benefit from a nutritional intervention. However, no such screening tools have been validated to identify older hospitalized patients for which dietary counseling, ONS or a combination of both might be beneficial [52, 54].

### Strengths and limitations

This systematic review and meta-analysis has several limitations that should be considered. First, only 11 studies were included. This might be a result of our strict inclusion criteria—that is, only RCT studies and readmission as the sole outcome. On the one hand, the limited number of studies might affect the external validity of this study. On the other hand, strict inclusion criteria often improve homogeneity, thus making a meta-analysis more feasible. In addition, we included studies from different continents (i.e., Europe, North America and Australia); thus, our results might be transferable to older patients living in other countries with a similar health-care system as the included countries.

Overall, the included studies had a low-to-medium risk of bias when assessing the quality but with the lack of blinding as an important exception. The difficulty in blinding the participants and health professionals delivering the interventions lowers the methodological quality and increases the risk of performance and detection bias. This is a well-known challenge in both nutritional and physical exercise interventions and poses a threat to the internal validity of the present review. Furthermore, the included studies had varying definitions of readmission (from 28 days to 90 days), and readmission was the primary outcome measure in only three of the studies. Also, most of the studies compared interventions to usual care, however, what constituted usual care was seldom described. The sample sizes of the included interventions were highly variable (ranging from 35 to 622), thus highlighting the overall lack of large-scale empirical research. Moreover, we have not mapped or analyzed reasons for hospital readmission. This might be a limitation since risk for readmission might vary between different diagnosis. Future studies should include such data and perform subgroup analysis if possible.

A strength of the present study is the comprehensive attempt to collate and evaluate the evidence for the effects of both physical exercise and nutritional interventions on readmission in older patients.

### Implications for policy

The share of the world’s population aged 65 years or older is approximately 9% but is higher in North America and Central Asia (16–17%) [55]. Still, the majority of the population admitted to hospital is within this age group—for example, in England, in the period 2015–2016, more than 40% of the admitted patients were 65 years or older [56]. Up to 25% experience being readmitted to hospital; therefore, it is important to identify interventions and components that may reduce hospital readmission to reduce both personal costs and stress for the patient, as well as health-care costs and pressure on the health-care system.

## Conclusion

This meta-analysis establishes an important quantitative framework for understanding the effects of physical exercise and nutritional interventions on readmission in older patients. This study suggests that nutritional interventions in accordance with international guidelines can potentially reduce readmission rates in older patients. Furthermore, this study sheds light on the need for future high-quality RCTs regarding the effects of physical exercise and nutritional interventions on readmission in older patients.

## Supplementary Information

**Additional file 1.**

**Additional file 2.**

## Data Availability

The datasets used and/or analyzed during the current study are available from the corresponding author on reasonable request.

## Abbreviations

RCT: Randomized controlled trial
ONS: Oral nutritional supplement
