# Correction to: Effect of nutritional and physical exercise intervention on hospital readmission for patients aged 65 or older: a systematic review and meta-analysis of randomized controlled trials

**DOI:** 10.1186/s12966-021-01152-5

**Published:** 2021-06-25

**Authors:** Ellisiv Lærum-Onsager, Marianne Molin, Cecilie Fromholt Olsen, Asta Bye, Jonas Debesay, Christine Hillestad Hestevik, Maria Bjerk, Are Hugo Pripp

**Affiliations:** 1 Lovisenberg Diaconal University College, Oslo, Norway; 2 Department of Nursing and Health Promotion, Faculty of Health Sciences, Oslo Metropolitan University, Oslo, Norway; 3 Department of Health, Bjorknes University College, Oslo, Norway; 4 Department of Physiotherapy, Faculty of Health Sciences, Oslo Metropolitan University, Oslo, Norway; 5 Regional Advisory Unit for Palliative Care, Department of Oncology, Oslo University Hospital, Oslo, Norway; 6 Norwegian Institute of Public Health, Oslo, Norway; 7 Faculty of Health Sciences, Oslo Metropolitan University, Oslo, Norway; 8 Oslo Centre of Biostatistics and Epidemiology, Research Support Services, Oslo University Hospital, Oslo, Norway

**Correction to: Int J Behav Nutr Phys Act 18, 62 (2021)**

**https://doi.org/10.1186/s12966-021-01123-w**

Following the publication of the original article [1], the authors identified an error in Fig. 2. The correct figure is given below.

**Fig. 2:**
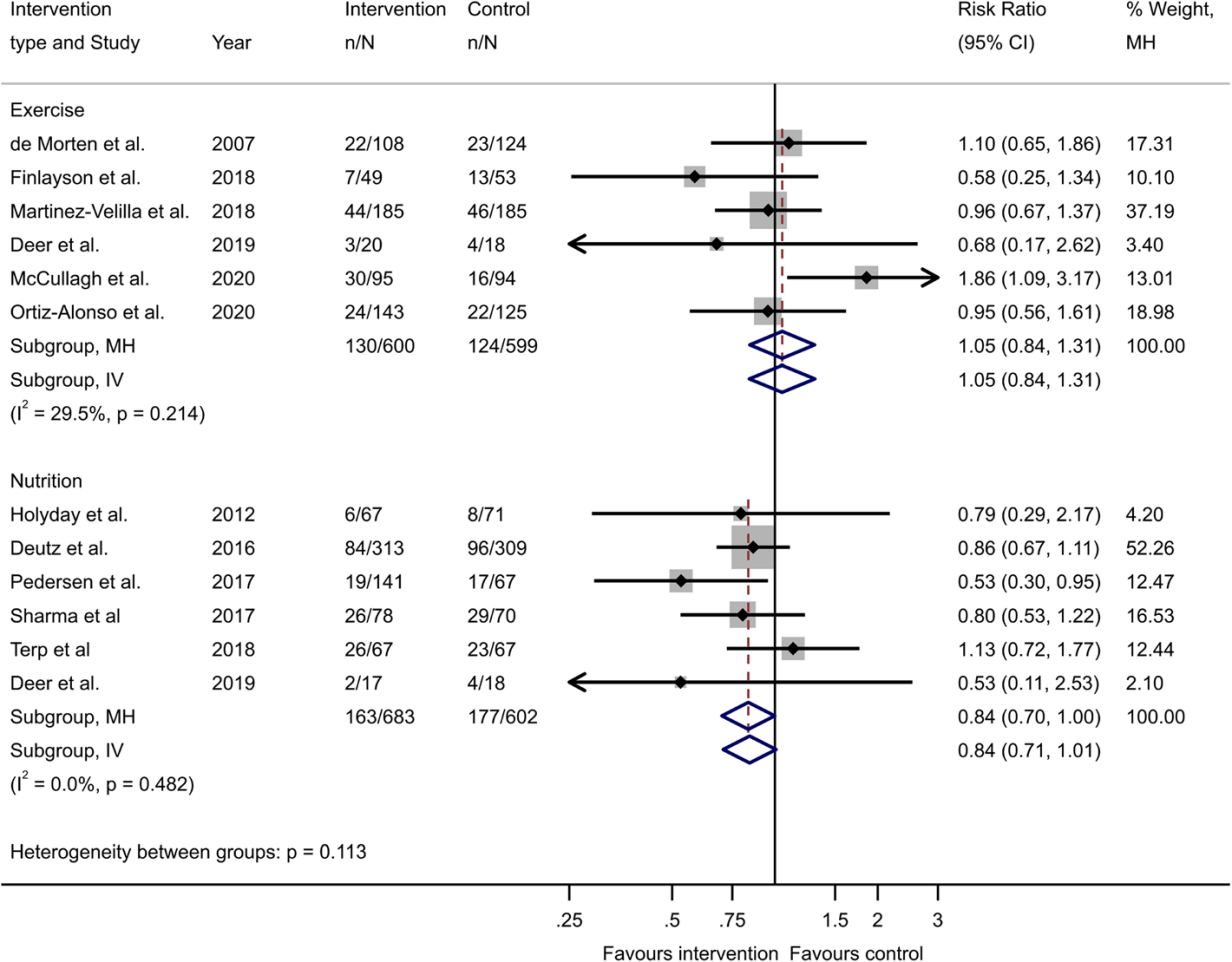
Pooled analysis presented as forest plots of the included studies on physical exercise and nutrition

The original article [1] has been corrected.
