# Supplementary material for: Effect of nutritional and physical exercise intervention on hospital readmission for patients aged 65 or older: a systematic review and meta-analysis of randomized controlled trials
Source: Int J Behav Nutr Phys Act. 2021 May 10;18:62. doi: 10.1186/s12966-021-01123-w (PMC8112053; doi:10.1186/s12966-021-01123-w)
Supplement: Supplementary file 1 — Additional file 1. [file 12966_2021_1123_MOESM1_ESM.pdf]

Search history

Database: Ovid MEDLINE(R) Epub Ahead of Print, In-Process & Other Non-Indexed Citations, Ovid MEDLINE(R) Daily and Ovid MEDLINE(R) 1946 to Present
Date: 18.05.2018, updated November 2018
Hits: 654

#	Searches	Results
1	Hip Fractures/	14000
2	Femoral Fractures/	15310
3	Femoral Neck Fractures/	8240
4	((hip or femur or femoral or trochanter* or subtrochanter* or intertrochanter* or pertrochanter* or acetatbulum) adj3 fracture*).tw,kw,kf.	36008
5	or/1-4	46927
6	physical therapy modalities/	33689
7	movement/	68193
8	gait/	24164
9	locomotion/	22939
10	Exercise Therapy/	34888
11	exercise/	91826
12	motor activity/	92338
13	Walking/	28384
14	Early Ambulation/	2549
15	rehabilitation/	17613
16	muscle strength/	15566
17	postural balance/	19931
18	resistance training/	6429
19	(exercise* or walking or training or retraining or mobili* or locomotion or gait or balanc* or physiotherap* or physio therap* or physical therap* or weight bearing or physical activit*).tw,kw,kf.	1171214
20	or/6-19	1366259
21	5 and 20	6421
22	AGED/	2778152
23	Frail Elderly/	9484
24	"AGED, 80 AND OVER"/	798955
25	(aged or elder or elders or elderly or old or older or olds or geriatric patient*).tw,kw,kf.	1772821
26	or/22-25	4055039
27	21 and 26	4604

28	controlled clinical trial/ or exp randomized controlled trial/	549854
29	controlled clinical trials as topic/ or exp randomized controlled trials as topic/	123246
30	Random Allocation/	94454
31	meta-analysis.pt.	88887
32	(random* or rct* or metaanaly* or meta analy* or quasiexperiment* or quasi experiment*).tw,kw,kf.	994487
33	or/28-32	1332798
34	27 and 33	654

Database: Embase Ovid 1980 to 2018 Week 18
Date: 18.05.2018, updated November 2018
Hits: 931

#	Searches	Results
1	hip fracture/	25061
2	acetabulum fracture/	2467
3	femoral head fracture/	82
4	femoral neck fracture/	981
5	femur intertrochanteric fracture/	1756
6	femur pertrochanteric fracture/	537
7	femur subtrochanteric fracture/	1365
8	femur trochanteric fracture/	1063
9	"fracture of greater trochanter"/	43
10	"fracture of lesser trochanter"/	13
11	((hip or femur or femoral or trochanter* or subtrochanter* or intertrochanter* or pertrochanter* or acetatbulum) adj3 fracture*).tw,kw.	44633
12	or/1-11	54324
13	physiotherapy/	76655
14	"movement (physiology)"/	32873
15	gait/	44976
16	walking/	58146
17	locomotion/	63325
18	physical activity/	126839
19	kinesiotherapy/	28474
20	exercise/	241633
21	motor activity/	43142
22	mobilization/	28308

23	rehabilitation/	68601
24	muscle training/	8407
25	muscle strength/	51550
26	resistance training/	13026
27	body equilibrium/	16261
28	(exercise* or walking or training or retraining or mobili* or locomotion or gait or balanc* or physiotherap* or physio therap* or physical therap* or weight bearing or physical activit*).tw,kw.	1458976
29	or/13-28	1766584
30	12 and 29	9779
31	limit 30 to aged <65+ years>	4687
32	(aged or elder or elders or elderly or old or older or olds or geriatric patient*).tw,kw.	2349306
33	30 and 32	4824
34	31 or 33	6491
35	meta analysis/	143944
36	randomization/	77999
37	randomized controlled trial/	499014
38	controlled clinical trial/	461416
39	(random* or rct* or metaanaly* or meta analy* or quasiexperiment* or quasi experiment*).tw,kw.	1315325
40	or/35-39	1665853
41	34 and 40	931

Database: AMED (Allied and Complementary Medicine) 1985 to May 2018
Date: 18.05.2018, updated november 2018
Hits: 64

#	Searches	Results
1	hip fractures/	576
2	femoral fractures/	125
3	Femoral neck fractures/	61
4	((hip or femur or femoral or trochanter* or subtrochanter* or intertrochanter* or petrochanter* or acetatbulum) adj3 fracture*).tw.	1041
5	or/1-4	1041
6	physical therapy modalities/	5376
7	mobilisation/	420
8	movement/	5226
9	locomotion/	745
10	Walking/	5183
11	gait/	2514

12	Weight bearing/	1453
13	exercise/	8884
14	exercise therapy/	7372
15	motor activity/	1521
16	rehabilitation/	53871
17	early ambulation/	73
18	balance/	3243
19	(exercise* or walking or training or retraining or mobili* or locomotion or gait or balanc* or physiotherap* or physio therap* or physical therap* or weight bearing or physical activit*).tw.	69477
20	or/6-19	107443
21	5 and 20	608
22	Aged/	13663
23	Frail elderly/	459
24	Aged 80/	927
25	(aged or elder or elders or elderly or old or older or olds or geriatric patient*).tw.	29527
26	or/22-25	29527
27	21 and 26	375
28	meta analysis/	248
29	randomized controlled trials/	1930
30	Random allocation/	314
31	(random* or rct* or metaanaly* or meta analy* or quasiexperiment* or quasi experiment*).tw.	17841
32	or/28-31	17996
33	27 and 32	64

Database: Cochrane
Date: 18.05.2018, updated 2018
Hits: 706

ID	Search	Hits
#1	MeSH descriptor: [Hip Fractures] this term only	1060
#2	MeSH descriptor: [Femoral Fractures] this term only	253
#3	MeSH descriptor: [Femoral Neck Fractures] this term only	354
#4	((hip or femur or femoral or trochanter* or subtrochanter* or intertrochanter* or pertrochanter* or acetatbulum) near/3 fracture*):ti,ab,kw	4258
#5	{or #1-#4}	4258
#6	MeSH descriptor: [Physical Therapy Modalities] this term only	3670
#7	MeSH descriptor: [Movement] this term only	2452
#8	MeSH descriptor: [Gait] this term only	1723
#9	MeSH descriptor: [Locomotion] this term only	340

#10	MeSH descriptor: [Exercise] this term only	13987
#11	MeSH descriptor: [Exercise Therapy] this term only	8977
#12	MeSH descriptor: [Motor Activity] this term only	3837
#13	MeSH descriptor: [Walking] this term only	3821
#14	MeSH descriptor: [Early Ambulation] this term only	365
#15	MeSH descriptor: [Rehabilitation] this term only	468
#16	MeSH descriptor: [Muscle Strength] this term only	3487
#17	MeSH descriptor: [Postural Balance] this term only	2284
#18	MeSH descriptor: [Resistance Training] this term only	2589
#19	(exercise* or walking or training or retraining or mobili* or locomotion or gait or balanc* or physiotherap* or "physio therap*" or "physical therap*" or "weight bearing" or "physical activit*"):ti,ab,kw	139962
#20	{or #6-#19}	143162
#21	MeSH descriptor: [Aged] this term only	618
#22	MeSH descriptor: [Frail Elderly] this term only	695
#23	MeSH descriptor: [Aged, 80 and over] this term only	161
#24	(aged or elder or elders or elderly or old or older or olds or "geriatric patient*"):ti,ab,kw	448022
#25	{or #21-#24}	448022
#26	{and #5, #20, #25}	706

Database: Cinahl (Ebscohost)
Dato: 18.05.2018, updated november 2018
Hits: 64

#	Query	Results
S1	(MH "Hip Fractures")	5,190
S2	(MH "Femoral Fractures")	2,033
S3	TI (((hip or femur or femoral or trochanter* or subtrochanter* or intertrochanter* or pertrochanter* or acetatbulum) N2 fracture*)) OR AB (((hip or femur or femoral or trochanter* or subtrochanter* or intertrochanter* or pertrochanter* or acetatbulum) N2 fracture*))	6,962
S4	S1 OR S2 OR S3	9,031
S5	(MH "Physical Therapy")	25,471
S6	(MH "Movement")	7,337
S7	(MH "Gait")	4,920
S8	(MH "Gait Training")	1,496
S9	(MH "Locomotion")	807
S10	(MH "Exercise")	29,286
S11	(MH "Therapeutic Exercise")	14,822

S12	(MH "Motor Activity")	4,969
S13	(MH "Walking")	13,112
S14	(MH "Early Ambulation")	656
S15	(MH "Rehabilitation")	12,314
S16	(MH "Muscle Strength")	12,172
S17	(MH "Muscle Strengthening")	9,991
S18	(MH "Resistance Training")	2,009
S19	(MH "Weight-Bearing")	3,384
S20	(MH "Balance Training, Physical")	825
S21	TI ((exercise* or walking or training or retraining or mobili* or locomotion or gait or balanc* or physiotherap* or “physio therap*” or “physical therap*” or “weight bearing” or “physical activit*”)) OR AB ((exercise* or walking or training or retraining or mobili* or locomotion or gait or balanc* or physiotherap* or “physio therap*” or “physical therap*” or “weight bearing” or “physical activit*”))	256,723
S22	S5 OR S6 OR S7 OR S8 OR S9 OR S10 OR S11 OR S12 OR S13 OR S14 OR S15 OR S16 OR S17 OR S18 OR S19 OR S20 OR S21	313,079
S23	(MH "Aged") OR (MH "Aged, 80 and Over")	429,221
S24	TI ((aged or elder or elders or elderly or old or older or olds or geriatric patient*)) OR AB ((aged or elder or elders or elderly or old or older or olds or geriatric patient*))	243,459
S25	S23 OR S24	549,052
S26	S4 AND S22 AND S25	1,104
S27	(MH "Randomized Controlled Trials")	42,132
S28	TI ((random* or rct* or metaanaly* or "meta analy*" or quasiexperiment* or "quasi experiment*")) OR AB ((random* or rct* or metaanaly* or "meta analy*" or quasiexperiment* or "quasi experiment*"))	183,089
S29	S27 OR S28	193,436
S30	S26 AND S29	214

Database: Web of Science (Core collection: Indexes=SCI-EXPANDED, ESCI)
Dato: 22.05.2018, updated november 2018
Hits: 1265

Set	Searches	Results
# 5	#4 AND #3 AND #2 AND #1<br>Indexes=SCI-EXPANDED, ESCI Timespan=All years	1,265
# 4	TS=((random* or rct* or metaanaly* or "meta analy*" or quasiexperiment* or "quasi experiment*"))<br>Indexes=SCI-EXPANDED, ESCI Timespan=All years	1,593,406
# 3	TS=((aged or elder or elders or elderly or old or older or “geriatric patient*”))<br>Indexes=SCI-EXPANDED, ESCI Timespan=All years	2,961,620

# 2	TS=((exercise* or walking or training or retraining or mobili* or locomotion or gait or balanc* or physiotherap* or “physio therap*” or “physical therap*” or “weight bearing” or “physical activit*” or "motor activit*"))<br>Indexes=SCI-EXPANDED, ESCI Timespan=All years	1,683,444
# 1	TS=(((hip or femur or femoral or trochanter* or subtrochanter* or intertrochanter* or pertrochanter* or acetatbulum) NEAR/2 fracture*))<br>Indexes=SCI-EXPANDED, ESCI Timespan=All years	35,884

Database: Pedro
Dato: 23.05.2018, updated November 2018
Hits: 53 + 157 + 14 + 50 + 4 + 25 (many duplicates)
Hip fracture AND systematic review: 53
Hip fracture AND clinical trial: 157
Femoral fracture AND systematic review: 14
Femoral fracture AND clinical trial: 50
Femur fracture AND systematic review: 4
Femur fracture AND clinical trial: 25
