# Supplementary material for: Effect of nutritional and physical exercise intervention on hospital readmission for patients aged 65 or older: a systematic review and meta-analysis of randomized controlled trials
Source: Int J Behav Nutr Phys Act. 2021 May 10;18:62. doi: 10.1186/s12966-021-01123-w (PMC8112053; doi:10.1186/s12966-021-01123-w)
Supplement: Supplementary file 2 — Additional file 2. [file 12966_2021_1123_MOESM2_ESM.pdf]

Search history: UPDATE SEARCH

The searches are repeated exactly as in the original search, but there is a time limit set: 2018-2020, to capture the latest articles. Among the references identified in the update search, there will be references that have already been identified, this because the original search was performed in November 2018. The period January 2018-November 2018 is covered by both searches.

Database: Ovid MEDLINE(R) and Epub Ahead of Print, In-Process & Other Non-Indexed Citations and Daily <1946 to November 14>
Date: 06.03.2020
Hits: 403

#	Searches	Results
1	AGED/	3020280
2	"AGED, 80 AND OVER"/	891538
3	Frail Elderly/	11037
4	(aged or elder or elders or elderly or old or older or olds or geriatric patient*).tw,kw,kf.	1974916
5	or/1-4	4446331
6	Patient Readmission/	16532
7	(readmission* or re admission or rehosp* or re hosp* or readmit* or re admit*).tw,kw,kf.	37680
8	or/6-7	41754
9	exp Physical Therapy Modalities/	149876
10	exp MOVEMENT/	547630
11	GAIT/	27126
12	LOCOMOTION/	24996
13	exp Exercise Therapy/	49260
14	exp EXERCISE/	189828
15	Motor Activity/	95889
16	Early Ambulation/	2817
17	REHABILITATION/	18107
18	Muscle Strength/	19083
19	Postural Balance/	22598
20	exp Physical Fitness/	29078
21	Physical exertion/	56148
22	exp Physical Endurance/	32927
23	exp Diet/	274556
24	Meals/	2888
25	Eating/	52048
26	Food/	32585
27	exp Nutrition therapy/	99648
28	Nutrition assessment/	14696
29	Elder nutritional physiological phenomena/	222
30	exp Nutritional physiological phenomena/	584158

31	(exercise* or walking or training or retraining or mobili* or locomotion or gait or balanc* or physiotherap* or physio therap* or physical therap* or weight bearing or physical activit* or physical fitness or physical endurance or physical exertion or muscle strength or diet* or nutrition* or meal* or eating or food* or calorie intake).tw,kw,kf.	2378849
32	or/9-31	3060458
33	controlled clinical trial/ or exp randomized controlled trial/	590601
34	controlled clinical trials as topic/ or exp randomized controlled trials as topic/	138687
35	Random Allocation/	102229
36	meta-analysis.pt.	111566
37	(random* or rct* or metaanaly* or meta analy* or quasiexperiment* or quasi experiment*).tw,kw,kf.	1219527
38	or/33-37	1539425
39	and/5,8,32,38	496
40	limit 39 to yr="2018 -Current"	117

Database: Embase
Date: 06.03.2020
Hits: 186

#	Searches	Results
1	aged/	2908066
2	frail elderly/	9883
3	very elderly/	188634
4	(aged or elder or elders or elderly or old or older or olds or geriatric patient*).tw,kw.	2747238
5	or/1-4	4902637
6	hospital readmission/	60848
7	(readmission* or re admission* or rehosp* or re hosp* or readmit* or re admit*).tw,kw.	71355
8	or/6-7	84389
9	exp physiotherapy/	83753
10	"movement (physiology)"/	32404
11	locomotion/	69595
12	exp physical activity/	406203
13	exp exercise/	330456
14	exp physical capacity/	40197
15	exp kinesiotherapy/	75729
16	motor activity/	43930
17	exp mobilization/	31416
18	rehabilitation/	75738
19	muscle strength/	61067
20	resistance training/	17214
21	body equilibrium/	18083
22	fitness/	36526
23	endurance/	23450
24	or/9-23	1047219

25	exp diet therapy/	336996
26	exp nutrition/	2114445
27	exp food intake/	323330
28	or/25-27	2201785
29	(exercise* or walking or training or retraining or mobili* or locomotion or gait or balanc* or physiotherap* or physio therap* or physical therap* or weight bearing or physical activit* or physical fitness or physical endurance or physical exertion or muscle strength or diet* or nutrition* or meal* or eating or food* or calorie intake).tw,kw.	3006327
30	or/24,28-29	4604119
31	controlled clinical trial/	463519
32	randomized controlled trial/	593587
33	meta analysis/	182247
34	randomization/	86205
35	(random* or rct* or metaanaly* or meta analy* or quasiexperiment* or quasi experiment*).tw,kw.	1657299
36	or/31-35	1971953
37	and/5,8,30,36	768
38	limit 37 to yr="2018 -Current"	186

Database: AMED (Allied and Complementary Medicine) <1985 to November 2018>
Date: 06.03.2020
Hits: 3

#	Searches	Results
1	Aged/	15338
2	aged 80/	1256
3	Frail elderly/	508
4	(aged or elder or elders or elderly or old or older or olds or geriatric patient*).tw.	33293
5	or/1-4	33293
6	(readmission* or re admission or rehosp* or re hosp* or readmit* or re admit*).tw.	361
7	exp physical therapy modalities/	28313
8	exp Kinematics/	32305
9	Rehabilitation/	59261
10	exp Exercise/	9741
11	Physical fitness/	2613
12	Physical endurance/	720
13	exp Nutrition/	4266
14	Diet therapy/	2026
15	Food/	780
16	eating/	202
17	(exercise* or walking or training or retraining or mobili* or locomotion or gait or balanc* or physiotherap* or physio therap* or physical therap* or weight bearing or physical activit* or physical fitness or physical endurance	91035

	or physical exertion or muscle strength or diet* or nutrition* or meal* or eating or food* or calorie intake).tw.	
18	or/7-17	143692
19	Meta analysis/	313
20	Randomized controlled trials/	2377
21	Random allocation/	335
22	(random* or rct* or metaanaly* or meta analy* or quasiexperiment* or quasi experiment*).tw.	22018
23	or/19-22	22018
24	and/5-6,18,23	11
25	limit 24 to yr="2018 -Current"	3

Database: Cochrane library
Date: 06.03.2020
Hits: 665

#1	MeSH descriptor: [Aged] this term only	630
#2	MeSH descriptor: [Aged, 80 and over] this term only	156
#3	MeSH descriptor: [Frail Elderly] this term only	682
#4	(aged or elder or elders or elderly or old or older or olds or geriatric patient*):ti,ab,kw	551521
#5	{or #1-#4}	551521
#6	MeSH descriptor: [Patient Readmission] this term only	1005
#7	(readmission* or re admission or rehosp* or re hosp* or readmit* or re admit*):ti,ab,kw	12107
#8	{or #6-#7}	12107
#9	MeSH descriptor: [Physical Therapy Modalities] explode all trees	24179
#10	MeSH descriptor: [Movement] explode all trees	30362
#11	MeSH descriptor: [Gait] this term only	1811
#12	MeSH descriptor: [Locomotion] this term only	332
#13	MeSH descriptor: [Exercise Therapy] explode all trees	12905
#14	MeSH descriptor: [Exercise] explode all trees	23202
#15	MeSH descriptor: [Motor Activity] this term only	3664
#16	MeSH descriptor: [Early Ambulation] this term only	365
#17	MeSH descriptor: [Rehabilitation] this term only	308
#18	MeSH descriptor: [Muscle Strength] this term only	4049
#19	MeSH descriptor: [Postural Balance] this term only	2544
#20	MeSH descriptor: [Physical Fitness] explode all trees	3073
#21	MeSH descriptor: [Physical Endurance] this term only	3200
#22	MeSH descriptor: [Diet] explode all trees	17645

#23	MeSH descriptor: [Meals] this term only	635
#24	MeSH descriptor: [Eating] this term only	2609
#25	MeSH descriptor: [Food] this term only	1244
#26	MeSH descriptor: [Nutrition Therapy] explode all trees	9022
#27	MeSH descriptor: [Nutrition Assessment] this term only	662
#28	MeSH descriptor: [Elder Nutritional Physiological Phenomena] this term only	33
#29	MeSH descriptor: [Nutritional Physiological Phenomena] explode all trees	29301
#30	(exercise* or walking or training or retraining or mobili* or locomotion or gait or balanc* or physiotherap* or physio therap* or physical therap* or weight bearing or physical activit* or physical fitness or physical endurance or physical exertion or muscle strength or diet* or nutrition* or meal* or eating or food* or calorie intake):ti,ab,kw	342307
#31	{or #9-#30}	354285
#32	{and #5, #8, #31}	1377
	with Cochrane Library publication date from Jan 2018 to Mar 2020	665

NOTABENE: The updated search in the Cochrane library identified 325 articles with a publication date from 1999-2018, these were removed from the EndNote library before duplicate checks were performed. The total number of articles identified from Cochrane was therefore 340.

Database: Cinahl with full text, via Ebscohost
Date: 09.03.2020
Hits: 68

#	Query	Results
S1	(MH "Aged")	739,703
S2	(MH "Aged, 80 and Over")	272,571
S3	(MH "Frail Elderly")	6,949
S4	TI ((aged or elder or elders or elderly or old or older or olds or "geriatric patient*")) OR AB ((aged or elder or elders or elderly or old or older or olds or "geriatric patient*"))	424,990
S5	S1 OR S2 OR S3 OR S4	992,899
S6	(MH "Readmission")	12,308
S7	TI ((readmission* or "re admission" or rehosp* or "re hosp*" or readmit* or "re admit*")) OR AB ((readmission* or "re admission" or rehosp* or "re hosp*" or readmit* or "re admit*"))	16,953
S8	S6 OR S7	21,010
S9	(MH "Physical Therapy+")	128,585
S10	(MH "Movement+")	103,021
S11	(MH "Gait Training+")	1,802

S12	(MH "Motor Activity")	11,266
S13	(MH "Early Ambulation")	1,067
S14	(MH "Rehabilitation")	15,292
S15	(MH "Exercise+")	101,948
S16	(MH "Balance, Postural")	14,722
S17	(MH "Resistance Training")	4,601
S18	(MH "Rehabilitation, Geriatric")	2,796
S19	(MH "Physical Fitness")	15,974
S20	(MH "Nutrition+")	142,588
S21	(MH "Meals")	2,443
S22	(MH "Food")	12,806
S23	(MH "Eating")	6,059
S24	(MH "Nutrition Therapy (Iowa NIC)")	1
S25	(MH "Nutritional Status")	13,760
S26	(MH "Nutritional Assessment")	14,953
S27	(MH "Nutritional Support")	4,731
S28	(MH "Nutritional Physiology+")	126,403
S29	TI ((exercise* or walking or training or retraining or mobili* or locomotion or gait or balanc* or physiotherap* or "physio therap*" or "physical therap*" or "weight bearing" or "physical activit*" or "physical fitness" or "physical endurance" or "physical exertion" or "muscle strength" or diet* or nutrition* or meal* or eating or food* or "calorie intake") OR AB ((exercise* or walking or training or retraining or mobili* or locomotion or gait or balanc* or physiotherap* or "physio therap*" or "physical therap*" or "weight bearing" or "physical activit*" or "physical fitness" or "physical endurance" or "physical exertion" or "muscle strength" or diet* or nutrition* or meal* or eating or food* or "calorie intake"))	651,377
S30	S9 OR S10 OR S11 OR S12 OR S13 OR S14 OR S15 OR S16 OR S17 OR S18 OR S19 OR S20 OR S21 OR S22 OR S23 OR S24 OR S25 OR S26 OR S27 OR S28 OR S29	874,830
S31	(MH "Randomized Controlled Trials")	91,030
S32	TI ((random* or rct* or metaanaly* or "meta analy*" or quasiexperiment* or "quasi experiment*")) OR AB ((random* or rct* or metaanaly* or "meta analy*" or quasiexperiment* or "quasi experiment*"))	362,223
S33	S31 OR S32	381,019
S34	S5 AND S8 AND S30 AND S33	240
S35	S5 AND S8 AND S30 AND S33<br>Limiters - Published Date: 20180101-20201231	68

Database: Food Science Source, via Ebscohost
Date: 09.03.2020
Hits: 4

#	Query	Results
S1	DE "OLDER people"	2,280
S2	TI ((aged or elder or elders or elderly or old or older or olds or "geriatric patient*")) OR AB ((aged or elder or elders or elderly or old or older or olds or "geriatric patient*"))	106,532
S3	S1 OR S2	106,726
S4	DE "PATIENT readmissions"	326
S5	TI ((readmission* or "re admission" or rehosp* or "re hosp*" or readmit* or "re admit*")) OR AB ((readmission* or "re admission" or rehosp* or "re hosp*" or readmit* or "re admit*"))	950
S6	S4 OR S5	998
S7	DE "PHYSICAL therapy"	327
S8	DE "LOCOMOTION"	225
S9	DE "EXERCISE"	6,922
S10	DE "EXERCISE therapy"	467
S11	DE "WALKING"	1,662
S12	DE "EARLY ambulation (Rehabilitation)" OR DE "REHABILITATION"	613
S13	DE "RESISTANCE training" OR DE "PHYSICAL training & conditioning"	722
S14	DE "FUNCTIONAL training" OR DE "PHYSICAL training & conditioning" OR DE "ACTIVITIES of daily living training" OR DE "ACTIVITIES of daily living"	974
S15	DE "PHYSICAL fitness" OR DE "PHYSICAL fitness for older people" OR DE "PHYSICAL activity"	12,302
S16	DE "DIET"	24,189
S17	DE "NUTRITION"	34,749
S18	DE "DIET therapy"	3,020
S19	DE "NUTRITIONAL assessment"	4,961
S20	DE "NUTRITIONAL status"	3,392
S21	TI ((exercise* or walking or training or retraining or mobili* or locomotion or gait or balanc* or physiotherap* or "physio therap*" or "physical therap*" or	584,503

	"weight bearing" or "physical activit*" or "physical fitness" or "physical endurance" or "physical exertion" or "muscle strength" or diet* or nutrition* or meal* or eating or food* or "calorie intake") OR AB ((exercise* or walking or training or retraining or mobili* or locomotion or gait or balanc* or physiotherap* or "physio therap*" or "physical therap*" or "weight bearing" or "physical activit*" or "physical fitness" or "physical endurance" or "physical exertion" or "muscle strength" or diet* or nutrition* or meal* or eating or food* or "calorie intake"))	
S22	S7 OR S8 OR S9 OR S10 OR S11 OR S12 OR S13 OR S14 OR S15 OR S16 OR S17 OR S18 OR S19 OR S20 OR S21	595,440
S23	DE "RANDOMIZED controlled trials"	12,394
S24	TI ((random* or rct* or metaanaly* or "meta analy*" or quasiexperiment* or "quasi experiment*")) OR AB ((random* or rct* or metaanaly* or "meta analy*" or quasiexperiment* or "quasi experiment*"))	100,535
S25	S23 OR S24	101,661
S26	S3 AND S6 AND S22 AND S25	14
S27	S3 AND S6 AND S22 AND S25<br>Limiters - Publication Date: 20180101-20201231	4

Database: Web of Science, core collection
Date: 09.03.2020
Hits: 159

Set	Searches	Results
#6	#4 AND #3 AND #2 AND #1<br>**Refined by: PUBLICATION YEARS:** (2020 OR 2019 OR 2018)<br>*Indexes=SCI-EXPANDED, SSCI, A&HCI, ESCI Timespan=1987-2020*	159
# 5	#4 AND #3 AND #2 AND #1<br>Indexes=SCI-EXPANDED, SSCI, A&HCI, ESCI Timespan=1987-2018	546
# 4	TS=(random* or rct* or metaanaly* or "meta analy*" or quasiexperiment* or "quasi experiment*")<br>Indexes=SCI-EXPANDED, SSCI, A&HCI, ESCI Timespan=1987-2018	1,895,611
# 3	TS=(exercise* or walking or training or retraining or mobili* or locomotion or gait or balanc* or physiotherap* or "physio therap*" or "physical therap*" or "weight bearing" or "physical activit*" or "physical Fitness" or "physical endurance" or "physical exertion" or	4,554,385

	"muscle strength" or "motor activit*" or movement or moving or diet* or nutrition* or meal* or eating or food* or "calorie intake")<br>Indexes=SCI-EXPANDED, SSCI, A&HCI, ESCI Timespan=1987-2018	
# 2	TS=(readmission* or "re admission" or rehosp* or "re hosp*" or readmit* or "re admit*")<br>Indexes=SCI-EXPANDED, SSCI, A&HCI, ESCI Timespan=1987-2018	38,993
# 1	TS=(aged or elder or elders or elderly or old or older or olds or "geriatric patient*")<br>Indexes=SCI-EXPANDED, SSCI, A&HCI, ESCI Timespan=1987-2018	3,874,906
